# MicroRNA in Sjögren's Syndrome: Their Potential Roles in Pathogenesis and Diagnosis

**DOI:** 10.1155/2018/7510174

**Published:** 2018-06-07

**Authors:** M. Reale, C. D'Angelo, E. Costantini, M. Laus, A. Moretti, A. Croce

**Affiliations:** ^1^Department of Medical, Oral and Biotechnological Sciences, Unit of Immunodiagnostic and Molecular Pathology, “G. d'Annunzio” University, Via Dei Vestini 31, 66100 Chieti, Italy; ^2^Department of Medical, Oral and Biotechnological Sciences, Otorhinolaryngology Clinic, Clinical Hospital SS. Annunziata, “G. d'Annunzio” University, Via Dei Vestini 31, 66100 Chieti, Italy

## Abstract

Sjögren's syndrome (SS) or sicca syndrome was described by Swedish ophthalmologist Sjögren in the year 1933 for the first time. The etiology of the SS is multifunctional and includes a combination of genetic predisposition and environmental as well as epigenetic factors. It is an autoimmune disease characterized by features of systemic autoimmunity, dysfunction, and inflammation in the exocrine glands (mainly salivary and lacrimal glands) and lymphocytic infiltration of exocrine glands. In fact, the involvement of lacrimal and salivary glands results in the typical features of dry eye and salivary dysfunction (xerostomia). Only in one-third of the patients also present systemic extraglandular manifestations. T cells were originally considered to play the initiating role in the autoimmune process, while B cells were restricted to autoantibody production. In recent years, it is understood that the roles of B cells are multiple. Moreover, autoantibodies and blood B cell analysis are major contributors to a clinical diagnosis of Sjögren's syndrome. Recently, there has been rising interest in microRNA implication in autoimmunity. Unfortunately, to date, there are only a few studies that have investigated their participation in SS etiopathogenesis. The purpose of this work is to gather the data present in the literature to clarify this complex topic.

## 1. Introduction

### 1.1. Sjögren's Syndrome

Sjögren's syndrome (SS) is an autoimmune disease characterized by lymphocytic infiltration of salivary and lacrimal glands that results in eye and mouth dryness [1]. The SS prevalence is approximately 3% of the worldwide adults and has been reported to rarely affect children [2]. However, epidemiological studies underline the marked predilection for female, with a ratio of 9 : 1 to male, with age between 20 and 50 years [3]. The disorder was described by Mikulicz in 1892, but only in 1933, Dr. Henrik Sjögren published an article on a cluster of women presenting keratoconjunctivitis sicca, lymphoid infiltrations of the conjunctiva, cornea, lacrimal and parotid glands, a history of arthritis, and swelling of the salivary glands, in order to distinguish the SS from xerophthalmia [4].

SS is a multifactorial syndrome, involving environmental factors, genetic predisposition, and hormonal factors in the presence of the innate and acquired immune system costimulation [2, 5]. Although the pathogenesis of SS remains largely unknown, the autoimmunity is considered to be the key player in the syndrome development.

SS may occur alone as the primary SS (pSS) or as the secondary SS (sSS), in association with other autoimmune diseases such as systemic lupus erythematosus (SLE) or rheumatoid arthritis (RA) [1]. The pSS is characterized by xerophthalmia, xerostomia, xerosis, and systemic extraglandular organ involvement [6]. The prognosis for pSS is not favorable because the disease is linked to the onset of respiratory or kidney failure. The sSS is characterized by keratoconjunctivitis and xerostomia associated with other autoimmune disorders. The course of sSS depends strictly on the primary autoimmune pathology with an increase in tiredness and arthralgia [7].

Cytokine production, T lymphocytes, B-cell activating factor (BAFF), and autoantibodies secreted by B lymphocytes were found in the target tissue of SS and the salivary and lacrimal glands [8]. The histological analysis of glandular tissue reveals the presence of mononuclear lymphoid cell infiltration, replacing the glandular epithelium and causing epithelial destruction [2]. Glandular tissue destruction is observed in the minor salivary glands (MSG) with the massive presence of T and B cells [9]. Mononuclear cell infiltration is useful to classify SS severity. In mild lesions, CD4+ T cells are predominant, whereas in severe lesions B cells constitute the main population. The prevalence of CD4+ T cells decreases with lesion severity, whereas the prevalence of CD8+ T cells remains unchanged [9, 10]. More recently, the involvement of Th17 lymphocytes was detected, with a key role in inflammation, autoimmunity, and glandular tissue damage in SS [11]. Th17 cells, in association with Th1 and Th2 cells, are responsible for increased inflammatory cytokine production, such as IL-21 and IL-22 [12] which have been found in high concentration in the serum and salivary glands of SS patients [13, 14] with high relation to clinical symptoms. Moreover, matrix (interleukin) IL-17, transforming growth factor *β* (TGF-*β*), IL-6, and metalloproteinase (MMP) [11] are also expressed hypothesizing their involvement in the development and the onset of SS through the modulation of target tissue homeostasis and biological activities [13]. In mononuclear cells, infiltration has found also natural killer (NK) cells and professional antigen-presenting cells, such as macrophages and dendritic cells, and a small, but considerable, portion of the infiltrating mononuclear cells, and their percentage correlates with the grade of the lesions [9, 15, 16]. T regulatory cells (Tregs) also point out in MSG lesions, with increased expression in intermediate lesions. Treg cells were identified in an experimental animal model. Treg subset is important for their involvement in immune homeostasis [17], suppressive activity towards autoreactive lymphocytes, and release soluble mediators including IL-10 and TGF-*β* [18]. The lower number of Foxp3^+^ Tregs in SS lesions is in a relationship with the ineffective regulation of the inflammatory status that result in the loss of immune control and worsening of the state of illness [17].

B lymphocytes have the central role in the pathogenesis of SS; they stimulate the immune response against self- and nonself-antigens [19] through their overproduction. The affected exocrine glands are the major site of autoantibody formation [20, 21] for the local overexpression of B cells and BAFF [22, 23].

Several studies show the alteration in B cell subset in SS patients [19, 23, 24]; however, the mechanisms are still debated for the involvement of genetic, epigenetic, and environmental triggers promoting B cell activation. Recently, a genetic cohort study was conducted to evaluate new regions associated with SS, showing a correlation with the (major histocompatibility complex) MHC region and the presence of innate immune system pathway activation, the interferon regulatory factor 5 (IRF5), T cell activation (HLA and MHC associations, STAT4, IL12, KLRG1, SH2D2A, and NFAT5), and NF-*κ*B activation (TNIP1 and TNFAIP3) [24, 25]. Evidence on the environmental involvement in SS induction are supported by multiple factors, including ultraviolet light, smoking, or chemical exposure, that determine epigenetic change-associated SS. One of the principal alterations is the DNA demethylation with defective satellites and retrotransposons, splicing mutations, polymorphisms, and miRNA overexpression [26]. B cells' role in the pathogenesis of autoimmune diseases could be resumed in three key mechanisms: the production of inflammatory cytokines, autoantibody, and antigen-presenting cells. The autoantibody production represents the most important mechanism with the involvement of many different types of antibodies [24]. Elevated IgA level is a common finding and is strongly associated with abnormal salivary gland biopsy [27, 28], while IgG can be detected in half of the pSS patients, especially in those with extraglandular manifestations [28]. According to Tzioufas et al., these antibodies possess three different abilities: serving as disease markers, indicating the association with other autoimmune diseases, and exhibiting a possible pathogenetic role [29]. Some of these autoantibodies are against Ro/SSA (Sjögren's syndrome A) or La/SSB (Sjögren's syndrome B) ribonucleoprotein complexes. Anti-Ro/SSA antibodies represent two distinct entities of autoantibodies that react with two nonhomologous proteins, Ro52/TRIM21 and Ro60/TROVE2, respectively, a cytoplasmatic nuclear protein [30]. Ro52/TRIM21 contains a protein that acts as an intracellular Fc-Receptor, and it is implicated in the regulation of cell proliferation and activation, which induce cell death, as well as the regulation of TLR signaling and subsequent IFN [31]. Ro60/TROVE2 protein is a ring-shaped RNA-binding protein that participates in the quality control of nascent transcripts, including the recognition and leading of misfolded defective RNAs to degradation [32, 33]. The La/SSB autoantibodies are directed to a 47-kD protein that exists abundantly in both the nucleus and the cytoplasm. La protein is involved in RNA processing and metabolism. Detection of Ro/SSA and La/SSB autoantibodies occurs in 50–70% of patients [34]. Interestingly, the presence of anti-Ro/SSA is independent of anti-La/SSB; however, the coexpression is useful to identify SS patients [35]. The presence of these autoantibodies is related to a specific SS diagnosis, in particular in young patients with severe dysfunction in exocrine glands [35, 36]. Others autoantibodies with high prevalence in SS patients are rheumatoid factor, cryoglobulins, anticentromere antibodies (ACA), autoantibodies against cyclic citrullinated peptides (anti-CCP), calreticulin, antimitochondrial antibodies (AMA), antibodies to muscarinic receptors, autoantibodies targeting carbonic anhydrase II (anti-CAII), anti-smooth muscle antibodies (ASMA) [37], and antinuclear antibodies (ANA) are present in the sera of 59–85% of patients [38]. Other autoantibodies have been claimed to be specific for pSS diagnosis, such as antibodies to alpha-fodrin [39], muscarinic receptors [40], or Golgi [41], but their clinical relevance is still debated [30]. More recently, another set of autoantibodies has been detected in interleukin 14 alpha transgenic mouse (IL14*α*TG), an animal model for SS [42]. Antisalivary gland protein 1 (anti-SP1), anticarbonic anhydrase 6 (anti-CA6), and antiparotid secretory protein (anti-PSP) autoantibodies are found in SS patients also in the presence of Ro/SSA and La/SSB autoantibodies. SP-1, CA6, and PSP are considered early stage markers for SS [1] than anti-Ro and anti-La. Diagnosing SS resulted to be difficult for a wide range of the clinical spectrum of nonspecific symptoms and the most evidence related to other autoimmune diseases. For a definitive diagnosis of SS, the establishment of specific criteria is required. The main diagnostic criteria of SS are defined by the American College of Rheumatology-European League against Rheumatism in 2016 that has published a set [43, 44]. Dry mouth, dry eyes, circulating autoantibodies to Ro/SS-A and La/SS-B, and lymphocytic infiltration of salivary glands are the most common criteria for SS [1]. Detection of circulating antinuclear autoantibodies are identified in the sera of patients [45]. Studies in animal models and humans with SS demonstrate that SP-1, CA6, and PSP autoantibodies are expressed earlier in patients with lower scores in MGS biopsies [46–48]. These autoantibodies concentration became higher at the time of diagnosis [45, 49]. The identification of prediagnostic autoantibodies is related to an unfavorable diagnosis [49]. A correlation between novel autoantibodies and diagnosis has been demonstrated by Everett et al., in a specific cohort of SS patients with “idiopathic dry eyes.” The immune-mediated damage of lacrimal glands corresponds to anti-SP1, anti-CA6, and anti-PSP high presence in serum and represent markers for early Sjögren's syndrome diagnosis [47] Some authors showed that classical autoantibodies like ANAs, RF, anti-Ro/SSA, and anti La/SSB have a prognostic value for clinical diagnosis [45]. However other evidence is related to no association between these autoantibodies and the clinical and biological SS severity [48]. These data are supported by studies that define the MSG biopsy as the gold standard method for the detection of SS, also in 22-23% of seronegative patients [50, 51]. Further studies will be necessary to understand the role as well as the usefulness of antibodies and MSG biopsy in the diagnosis of SS.

During the last decade, genetic and epigenetic studies have been widely investigated in relation to SS and with the pathogenesis of other autoimmune disorders. In particular, the RNA interference system is a conserved biological response that regulates the expression of protein-coding genes. This mechanism represents an evolutionary system in experimental biology and may be important for comprehension in genomic and therapeutic interventions. This system involves microRNAs (miRNAs), molecules implicated in the control of several biologic processes, and autoimmune disease development [52, 53].

### 1.2. miRNA

miRNAs are 18 to 23 base pair (bp) noncoding RNAs that govern numerous biological processes regulating gene expression at the posttranscriptional level by degradation and translational repression of their targeted miRNAs.

It is estimated that human genome encodes up to one thousand miRNAs which are either transcribed as independent genes or embedded in the intronic region of other genes. The genes encoding miRNAs comprise 1 e 5% of all genes, making miRNAs the most abundant class of regulators that may control the expression of 30% of protein-coding genes. [54]. The inhibition of protein translation is achieved by a variety of mechanisms including direct cleavage of mRNAs or translational initiation repression and premature translation termination [55].

Beginning in the nucleus and ending in the cytoplasm, miRNA biogenesis is a complex process, which involves many steps. miRNAs are transcribed in the nucleus by RNA polymerase II as a longer preliminary transcript and are then generated by sequential processing by two RNase III enzymes, Drosha and Dicer. In the nucleus, the primary miRNA (pri-miRNA) transcripts are cleaved by Drosha into a 70-nucleotide stem-loop precursor referred as pre-miRNAs [56]. The pre-miRNA hairpin is exported by Exportin 5 to the cytoplasm and is further processed by Dicer into a double-stranded RNA, 19–24-nucleotide long, with one strand loaded in the RNA-induced silencing complex (RISC). At the core of the RISC ribonucleoprotein complex, duplex separation into single strands occurs, generating miRNAs. RISC consists in RNA helicase A and proteins such as argonaute 2 and TRBP that will facilitate the binding of miRNA to its mRNA target [57, 58]. For degradation or translational repression, mature miRNAs bind the 3′ untranslated region (UTR) of specific target miRNA with their so-called “seed” region. The seed is a sequence of nucleotides from position 2 to position 7 or 8 in the 5′ UTR region of the miRNA, responsible for the recognition and binding of the miRNA target [59]. For the subsequent regulatory action, if the complementary base pairing is perfect or near perfect, miRNA cleavage and degradation are induced. With incomplete base pairing, the resulting double-stranded RNAs lead to translational repression [60]. One-third of the transcriptome is suspected to posttranslational regulation by the 800–1000 human miRNAs since one miRNA can alter the expression of hundreds of miRNAs. miRNAs regulate different cellular processes such as embryonic development, cell differentiation, cell cycle and proliferation, apoptosis, immune cell development and immune responses, immune cell lineage commitment, and immune homeostasis [61, 62].

### 1.3. miRNA and Autoimmune Disease

To date, evidence from experimental animal models and clinical studies in humans have shown the involvement of miRNAs in the regulation of immune homeostasis [63–65]. Several miRNAs are reported to play a key role in immune cell development and differentiation of B and T cells from hematopoietic stem cells, for example, miR-155, miR-146, miR-132, and miR-181a and the cluster miR17–92 [64, 66].

It is understandable how a dysregulation in miRNA expression can be related to immune tolerance breakdown leading to autoimmune disease (AID) development. AID is a pathological condition in which autoantigens and inflammation affect one or more target organs, with cellular and tissue destruction [65, 67]. AIDs affect more than 3% and 80% of the worldwide population, with prevalence in female [68]. Dysregulation in miRNA expression can be found in several AIDs, for example, in rheumatoid arthritis (RA), type 1 diabetes mellitus (T1DM), multiple sclerosis (MS), Sjögren's syndrome (SS), systemic lupus erythematosus (SLE), inflammatory bowel disease (IBD), psoriasis (PS), primary biliary cirrhosis (PBC), and idiopathic thrombocytopenic purpura (ITP) [62, 67, 68]. MicroRNA expression profile has become widely studied as a potential biomarker of various autoimmune diseases like MS, RA, and SLE. Tregs of systemic lupus erythematosus mice exhibit altered regulatory phenotype and reduced suppressive capacity and Dicer expression, together with a distinct miRNA profile. miR-26a has been found decreased in experimental autoimmune encephalomyelitis mice and in multiple sclerosis patients acts as a regulator of the Th17/Treg cell balance, promoting the generation of Tregs and inhibiting the generation of Th17 [69]. miRNAs such as miR-15a, miR-15b, miR-181c, and miR-328 were downregulated in MS; contrarily, miR-15a, miR-19a, miR-22, miR-210, and miR-223 were upregulated in both regulatory T cells (Tregs) and other samples such as plasma, blood cells, PBMCs, and brain white matter tissues from MS patients.

Nevertheless, the miRNA translational silencing mechanism is highly complicated, since each miRNA is considered to target the suppression of several hundreds of mRNAs, whereas a single mRNA can be regulated by several distinct miRNAs that act cooperatively.

The exact mechanism by which miRNA expression can be altered is not completely understood, as well as their involvement in the AID pathogenesis.

Moreover, there are still a great number of contradictions concerning the interpretation of the obtained findings on the over- or underexpression of various miRNAs, considering also that miRNAs are unlikely to be unique to a particular disease process.

## 2. miRNA in Sjögren's Syndrome

In recent years, extensive research has been performed to characterize miRNAs and their regulation of immune responses and immune cell development.

An ever-increasing number of studies have reported that miRNAs are associated with SS salivary gland tissue inflammation and are shown to be deregulated in the SS salivary gland and also in long-term cultured salivary gland-derived epithelial cells and peripheral blood mononuclear cells from SS patients (Table 1).

In one of the first researches conducted to evaluate the possible presence of deregulated miRNA directly in the salivary glands of patients with SS, Alevizos et al. reported a reduced expression of miR-17-92 cluster [70]. miR-17, miR-18a, miR-19a, miR-20a, miR-19b-1, and miR-92a-1 are members of the miR-17-92 cluster that is important in the cell cycle, proliferation, apoptosis, and other pivotal processes and is often deregulated in cardiovascular, immune, and neurodegenerative diseases [71]. The decrease of the mir-17-92 cluster was associated with accumulation of mature B cells and pro-B cells with a marked reduction of pre-B events that have been linked to lymphoproliferative disease and autoimmunity [64, 66, 70].

Considering the research results available to date, miR-146a and miR-155 seem to be two miRNAs upregulated in response to the adaptative immune response in multiple cell types. miR-146a can be considered a key gene regulator for proinflammatory signaling; activated by NF-*κ*B, it acts as a negative feedback regulator of the immune response by targeting two genes TRAF6 (TNF receptor-associated factor 6) and IRAK1 (IL-1 receptor-associated kinase 1). Targeting the TNF receptor-associated factor 6 (TRAF6) and the IL-1 receptor-associated kinase (IRAK1), miR-146a controls the TLR/IFN pathway, the signal transducer and activator of transcription 1 (STAT1), and the interferon regulatory factor 5 (IRF5) [72, 73]. The posttranslational effect driven by miR-146a is the inhibition of IRAK1 and TRAF6 expression, impairing NF-*κ*B activity and reducing expression of NF-*κ*B target genes such as IL-6, IL-8, IL-1*β*, and TNF*α* proinflammatory cytokines. miR-146a has been found upregulated in PBMCs from patients affected by SS [74–77], even though this increased expression appeared correlated with IRAK1 underexpression and a contradictory overexpression of TRAF6 genes, from Zilahi et al. in 2011 [74]. miRNA-146a (miR-146a) is supposed to negatively regulate adaptative immunity, inflammatory response, and antiviral pathway, but it has to be stressed that the expression rate and suppressive effects of miR-146a, and of all types of miRNAs taken into consideration, could be different and varied depending on the specific tissue and disease.

Wang-Renault et al. in a recent study evaluated miRNA profile in purified T and B lymphocytes detecting increased expression levels of both miR-146a and miR-155 in T lymphocytes [78].

Examination of miR-155 targets reveals an effect on the response of Toll-like receptors and interleukin-1 receptors (TIRs) that are suspected to affect the immune response. Interestingly, the FoxP3 transcription factor, which is overexpressed in T cells infiltrating SS salivary glands, has been shown to induce miR-155 expression.

Two fold-increased level of miR-155 has been found also in cultured salivary gland epithelial cells from SS than controls [26].

miRNA expression is necessary for the development of Treg cells in the thymus and the efficient induction of Foxp3 by TGF-*β* in a cell-autonomous fashion. Foxp3 may directly activate several miRNAs such as miR-155, which is indispensable for Treg cells to normally respond to growth factor and largely dispensable for Treg cell suppressor function [79].

In PBMCs, Shi et al. confirm the overexpression of miR-146a level in patients with SS founding also a positive correlation with the score for parotid swelling and dry eyes [76]. However, the miR-155 expression level was significantly decreased in PBMCs from patients affected by SS, with a positive correlation with the score for dry eyes. Evidence shows the overactivation of B cells and T cells with the increased expression of miR-155 [64, 76, 80].

Williams et al., considering that monocytes and their derivatives of macrophages and DCs have abnormalities in autoimmune diseases such as SLE, RA, and SS, selected this cell subset in order to profile miRNAs in SS patients, focusing also on predicting their potential roles in SS pathogenesis. After the detection of deregulated miR-300, miR-609, miR-3162-3p, and miR-4701-5p, in SS patients compared to healthy donors, they pursued the pathways possibly influenced by these miRNAs, founding evidence on the targeting of TGF*β* signaling pathway. Also, MAPK, JNK, and p38 MAPK signaling pathways together with JAK-STAT signaling cascades, important for cytokine signaling, may be affected by SS-associated miRNAs. Therefore, proinflammatory cytokines and NF-*κ*B signaling pathways are presumed to be well maintained; SS-associated miRNAs seem to slope regulatory TGF*β* signaling responses, in SS monocytes [81].

miR-181a and miR-16, identified to be associated with Ro/SSA and La/SSB in patients with SS, were detected in labial salivary gland tissues showing a significant downregulation in patients with SS compared with the controls. A statistical correlation of miR-181a and miR-16 expression levels with SGPF scores revealed a higher level of miR-181a and miR-16 in patients with SS and high-grade inflammation SGPF scores, when compared with those patients exhibiting lower SGPF scores, suggesting that miR-181a and miR-16 may serve a role in the pathogenesis of SS [82]. Peng et al. reported that miR181a expression was elevated in PBMCs of patients with SS, with a positive correlation between miR-181a levels and ANA titer. Measurement of miR-181a levels in isolated B cells and T cells indicated that B cell population contribute in the major part to miR-181a expression probably explaining their compromised antigen sensitivity and then the dysfunction of exocrine glands in pSS [83]. miR-181a reduction also enhances the sensitivity of T cell response to antigens with high self-reactivity of the TCR [62].

Despite the uncertainty of the interpretation of the obtained results, in reviewing previous studies in this paper, the presence of deregulated miRNA in the SS appears clear. However, the mechanisms that regulate miRNA overexpression/underexpression and the understanding of its role in SS pathogenesis are still unknown. Larger SS patient cohorts need to be examined to determine if there could be a true and unique correlation between increased/decreased miRNA expression, the presence of SSA/SSB autoantibody reactivity, salivary gland inflammation, and biopsy focal score typical of SS.

## 3. Conclusions

The complexities of the miRNA network pathways, as well as the multiple targets of each miRNA, hinder the delineation of their role in SS disease phenomena. Of course, other miRNAs, epigenetic factors, as well as viral elements (including viral miRNAs) that possibly harbor in the affected tissues, might participate in the pathogenesis of this disease. To date, according to the SS criteria for diagnosis, labial salivary gland biopsy has the main importance, although this is an invasive procedure. Recently, with the increasing number of studies, revealing miRNA deregulation in PBMCs, sera, and saliva in SS, the identification of serum miRNA to mirror activation state of lymphocytic subsets may become an innovative tool to provide pivotal information about the nature of the immune responses occurring in autoimmune disease. However, the fact that serum miRNAs circulating in different compartments might provide an advantage, but since miRNAs may be released by all cells in the body and most of the blood miRNAs are released by organs and dividing cells, their specificity as diagnostic biomarkers is impacted by high background. Saliva is undoubtedly a conspicuous source of biomarkers in SS since it is the direct product of the affected target organ. Larger studies are necessary to validate salivary microRNAs as diagnostic markers in SS, but the ultimate goal would be to replace the invasive biopsies with less invasive methods. Moreover, novel diagnostic approaches may lead to a prompt diagnosis.

## Figures and Tables

**Table 1:** miRNA expression profiling in SS patients.

miRNA	*miRNA source*	Expression	Reference
miR-300, miR-609, miR-3162-3p, miR-4701-5p	Monocytes	Upregulated	(Williams et al. [81])
miR-181a	SG	Upregulated	(Kapsogeorgou et al. [53])
miR-200b	SGEC		
miR-223	PBMCs		
let-7b	SGEC	Downregulated	
miR-146a/b	PBMCs	Upregulated	(Zilahi et al. [74])
miR-17-92	MSG	Downregulated	(Alevizos and Illei [70])
miR-144-5p, miR-34a-5p, miR-425-3p/-5p, miR-145-5p, miR-21-3p, miR-18a-5p, miR-769-5p, miR-190a, miR-15a-5p, miR-106a-5p, miR-424-3p, miR-20b-5p, miR-16-1-3p, let-7e-5p, let-7d-5p, miR-126-3p/-5p, miR-186-5p, miR-20a-5p, miR-146a-5p, miR-484, miR-191-5p, miR-26a-5p, miR-222-3p	PBMCs	Upregulated	(Chen et al. [75])
miR-150-5p		Downregulated	
miR-155-5p, miR-222-3p, miR-146a-5p, miR-28-5p	T lymphocytes	Upregulated	(Wang-Renault et al. [78])
let-7d-3p, miR-30c-5p, miR-378a-3p		Downregulated	
miR-222-3p	B lymphocytes	Upregulated	
miR-378a-3p, miR-26a-5p, miR-30b-5p, miR-19b-3p		Downregulated	
miR-181a, miR-16	SG	Downregulated	(Wang et al. [82])
miR-146a	PBMCs	Upregulated	(Shi et al. [76])
miR-155		Downregulated	
miR-146a, miR-155	PBMCs	Upregulated	(Pauley et al. [77])
miR-181a	PBMCs	Upregulated	(Peng et al. [83])
miR-155, miR-181a	SGEC	Upregulated	(Le Dantec et al. [26])

SG: salivary glands; SGEC: cultured salivary glands epithelial cells; PBMCs: peripheral blood mononuclear cells; MSG: minor salivary glands.

## Abbreviations

SS: Sjögren's syndrome
miRNAs: MicroRNAs
ON: Optic neuritis
ANA: Antinuclear antibodies
ACA: Anticentromere antibodies
anti-CCP: Autoantibodies against cyclic citrullinated peptides
AMA: Antimitochondrial antibodies
anti-CAII: Antibodies to muscarinic receptor autoantibodies targeting carbonic anhydrase II
ASMA: Antismooth muscle antibodies
MSG: Minor salivary gland
SGB: Salivary gland biopsy
SGEC: Salivary gland epithelial cells.
